# The Process of Separating Bovine Serum Albumin Using Hydroxyapatite and Active Babassu Coal (*Orbignya martiana*)

**DOI:** 10.1155/2016/2808241

**Published:** 2016-06-07

**Authors:** Márcia Regina Ribeiro Alves, Abraham Damian Giraldo Zuñiga, Rita de Cássia Superbi Sousa, Carmelita Zacchi Scolforo

**Affiliations:** ^1^Biodiversity Network and Biotechnology Legal-Bionorte Amazon, Federal University of Tocantins, 109 Norte Avenida NS-15, ALCNO-14, Plan Director North, 77001-090 Palmas, TO, Brazil; ^2^Department of Chemistry, Federal University of Viçosa, Avenida PH Holfs, 36570-000 Viçosa, MG, Brazil; ^3^Department of Nutrition, Federal University of Espírito Santo, Avenida Fernando Ferrari, No. 514, Goiabeiras, 29075-010 Vitória, ES, Brazil

## Abstract

Bovine serum albumin is one of the major serum proteins; it plays an important role as a result of its functional and nutritional properties which have bioactive peptides. Adsorption method was used to separate protein, which involves hydroxyapatite, synthetic hydroxyapatite, and active babassu coal. Initially, characterization was carried out using the zeta potential of the adsorbents. Kinetic pseudo-first- and pseudo-second-order models were applied. For isotherms, equilibrium data studies were carried out using the Langmuir and Freundlich models, in addition to determining the efficiency of adsorptive process. The results of the zeta potential showed loads ranging from +6.9 to −42.8 mV. The kinetic data were better represented in the pseudo-second-order model with chemisorption characteristics. The adsorption capacity of the adsorbents decreased as pH increased, indicating that the electrostatic bonds and some functional groups of active babassu coal contributed to the reduction of adsorption, especially oxygen linked to carbon atoms. The value of pH 4.0 showed the best results of adsorption, being obtained as the maximum adsorption capacity (*q*_*m*_) and yield (%) (where *q*_*m*_ = 87.95 mg g^−1^ and 74.2%; 68.26 mg g^−1^ and 68.6%; and 36.18 mg g^−1^, 37.4%) of hydroxyapatite, synthetic hydroxyapatite, and active babassu coal, respectively.

## 1. Introduction

The world production of whey was estimated at 190 million tonnes per annum. Whey proteins generated approximately US$ 3.8 billion annually [1, 2]. Studies have been conducted with the aim of using serum with high added value. Nevertheless, whey is still considered a by-product of the dairy industry, despite being regarded as an important source of proteins with high nutritional value and biotechnological importance [3]. Unfortunately, it has a low economic value due to its misuse [4]. This by-product can be recovered for use in various industries [5, 6].

Bovine serum albumin (BSA) is one of the major serum proteins; it plays an important role as a result of its functional and nutritional properties which have bioactive peptides [7]. Its low cost compared to other proteins, wide availability, its structure, and functional similarity to human serum albumin enable its various biotechnological applications [8]. Based on these aspects, the separation of BSA is extremely important in biotechnology as it has contributed enough for use in adsorption studies of various surfaces [9]. The growth in this area resulted in an increase in large-scale surveys of protein purification process using various adsorbents. It is estimated that more than 60% of the total bioprocess is due to recovery and purification [10]. Two of the major challenges in the separation of whey proteins are the low concentration and the complexity of the serum. The use of alternative materials, such as hydroxyapatite and activated carbon, has been widely encouraged [10–13].

Hydroxyapatite (HA) is an adsorbent which has the formula Ca_10_(PO_4_)_6_(OH)_2_ and is considered a bioceramic widely used in the separation of biomolecules [9]. It is known as a synthetic alkaline calcium phosphate, stable at a wide pH and temperature range. It is the major mineral constituent of bone and teeth (~70%) in animals [9]. This adsorbent has a high affinity for proteins and has been used for the separation and purification processes of several proteins [14, 15].

Coal babassu (*Orbignya martiana*) is derived from a native palm of Northern Brazil, occupying large tracts of land with forest cover; it has a renewable resource of immense energy potential [16]. It is a carbonaceous porous material structure and has a small amount of heteroatoms, especially oxygen linked to carbon atoms. The active carbons are materials well known for their complex pore structure, high internal surface area, and good chemical stability and may have various functional groups containing oxygen on the surface [17].

In the separation process, some aspects influence the adsorption of proteins such as concentration, solution pH, interactions between molecules, and functional groups; therefore various studies have been made in recent years in order to examine the effect of such experimental conditions on protein adsorption [13]. Adsorption studies and bioactivity of BSA on hydroxyapatite were reported [11]. In this paper, characteristics such as concentration and different pH solutions and buffers were observed and the influence of BSA on the precipitation of calcium phosphate phases (CP) from simulated body fluid (SBF) was evaluated when the protein was previously bounded to HA surface. Swain and Sarkar [15] studied the adsorption of BSA on hydroxyapatite nanoparticles at different pH and temperature. Studies evaluated the effect of pH and temperature of BSA on hydroxyapatite [10]. A research with active babassu coal reported that interactions between functional groups and adsorbents decreased the adsorption capacity [12, 13]. Multiple interactions studies of hydrophilic and hydrophobic surfaces and electrostatic groups of loads which are present in buffer solutions were carried out [11, 13, 15, 18]. Note a dynamic complexity of the interactions of the protein and the surface of the adsorbent [19].

In this research, the analysis of interactions between adsorbents and protein was carried out according to the pH of the solutions and the loads encountered in the adsorbents, and the possible implications of these dynamic interactions between the adsorbents and protein were verified. The BSA was chosen as a model protein because it has been well utilized in adsorption studies, showing good stability, availability, purity, and high solubility in water. Hydroxyapatite was used due to high affinity with protein and the purity and availability of the adsorbent material. The active babassu coal was also used because of its availability, ability to add value to a by-product, and its underusage. During the study, characterization was made by zeta potential where charges were observed on the surface of the adsorbents. In addition, the equilibrium rate and adsorption were investigated and the behavior was analyzed according to the loads of protein and adsorbents. Isotherms of Langmuir and Freundlich were applied to determine the behavior in adsorption equilibrium. The adsorption was observed by kinetic model with nonlinear regression methods. The results obtained from the study will be used in further research of recovery of the whey proteins.

## 2. Materials and Methods

### 2.1. Samples and Reagents

Bovine serum albumin (BSA) and hydroxyapatite (HA), both with 98% purity, were purchased from Sigma Aldrich Chemical Co. The synthetic hydroxyapatite (HAS) was produced in the laboratory. The Active babassu coal (ACB) with a particle size of 1 to 2 mm was obtained from the company Tobasa Bioindustrial of Babaçu SA. For the fluid phase of ACB, throughout the study, a syringe filter (PTFE), hydrophilic with 0.45 *μ*m pore size and 25 mm diameter (analytical), was used. 0.01 mol L^−1^ sodium acetate and acetic acid of 0.01 mol L^−1^ were used for the preparation of buffer solutions. Calcium chloride, anhydrous dibasic sodium phosphate, sodium chloride, and sodium hydroxide were used for the preparation of synthetic hydroxyapatite. The solutions were prepared using Milli-Q water (resistivity = 18.2 MΩ cm). The experiments were conducted in the Laboratory of the Department of Chemistry, Federal University of Viçosa (UFV), Viçosa, MG, in partnership with the Federal University of Tocantins (UFT), Palmas, TO.

#### 2.1.1. Synthetic Hydroxyapatite (HAS)

The synthetic hydroxyapatite was prepared in the laboratory according to Sousa [20]. 500 mL of 0.5 mol L^−1^ CaCl_2_ and 500 mL of 0.5 mol L^−1^ Na_2_HPO_4_ solution at a flow rate of 250 mL h^−1^ were put in 1000 mL beaker containing 50 mL of NaCl of 1 mol L^−1^ and agitated at 210 rpm. The formation of a milky precipitate which was decanted and washed twice with deionized water was obtained. Then 25 mL NaOH 40% (w/v) was added, heated to boiling point, and maintained under stirring at 100 rpm/1 h. The precipitate formed was again decanted and washed three times with deionized water. Approximately 80 g of synthetic hydroxyapatite was obtained.

#### 2.1.2. Active Babassu Coal (ACB)

The active babassu coal (ACB) obtained was crushed in a Wiley mill (Quimis/Q298A21) to obtain smaller particles, and sieves of 100-mesh size were used. The charcoal was washed with deionized water and then the material was dried at 70°C/24 h (FANEM® model: 502) to be used later in the experiment.

### 2.2. Kinetics of Adsorption

The adsorption kinetics were investigated at room temperature (25 ± 1°C) and pH 4.0 and pH 7.0 for the three adsorbents (HA, HAS, and ACB). Samples of 0.0100 ± 0.0005 g (TKS-FA2004C scale) were preweighed in* Eppendorf* tubes of 13 of 2.0 mL. To each tube was added 800 *μ*L of buffer prepared and left under agitation at 20 rpm (brand DragonLab, MX-RDPro model) ±1 h. Following this was the addition of 1000 *μ*L of protein solution (3.0 mg mL^−1^) BSA. At predetermined time intervals (0 to 1400 min), the tubes were removed, and the solid phase of the fluid phase was separated by centrifugation (Hanil-Brand Model Smart R17) at 7500 g/15 min. An aliquot of the supernatant containing the nonadsorbed protein was removed from each tube at certain times for the quantification of BSA in fluid phase, using the spectrophotometric method of direct reading absorbance at 280 nm (PG Instruments Ltd., T80 + UV-VIS Spectrometer). Quantification of BSA was determined according to the calibration curve (1). In order to study the adsorption control mechanism of the process, such as mass transfer and chemical reaction, kinetic models were used to test experimental data according to (2), (3), and (4). The kinetic models (pseudo-first-order and pseudo-second-order) can be used where balance occurs [21, 22].

(1) q=vC0−Cm,

(2) dqtdt=k1qeq−qt,

(3) tqt=1k2qeq22+1qeq,

(4) h=k2·qe2,

where *q* is the concentration of protein adsorbed on the solid phase (g mg^−1^), *C*_0_ and *C* represent the initial and equilibrium concentrations (mg mL^−1^), *V* is the solution volume (mL), *m* is the mass of the adsorbent material (g), *k*_1_ is the rate constant for pseudo-first-order model of adsorption (min^−1^), and *q* and *q*_*t*_ denote the amounts of protein adsorbed and the equilibrium time *t* (mg g^−1^), respectively. *k*_2_ (mg g^−1^ min^−1^) is the constant of pseudo-second-order rate in the adsorption process. The constant *k*_2_ is used to determine the initial adsorption rate *h* (min^−1^) for *t* → 0.

### 2.3. Quantification of Separation Efficiency (%) of Bovine Serum Albumin

The same procedure of adsorption kinetics was used for the three adsorbents prepared with initial concentration of 3.0 mg mL^−1^/24 h period. The supernatant was removed in the experiment for direct reading in spectrophotometer. From the absorbance values of the solutions read at the spectrophotometer from the calibration curve, the adsorption capacity of each adsorbent was determined using (5). The separation efficiency of the adsorption process (Efic) was obtained from *C*_0_ which is the initial concentration (mg mL^−1^); *C* is the final concentration (mg mL^−1^) in equilibrium, and *V* is the solution volume (mL):

(5) $$\begin{array}{c} Efic\left(\;\%\;\right)=\frac{{VC}_{0}-VC}{VC_{0}}\times 100. \end{array}$$

### 2.4. Zeta Potential (*Zp*)

The zeta potential study was used to quantify the loading surface of the adsorbents (HA, HAS, and ACB) at a concentration of 1.0 mg mL^−1^ and 25°C for each of the pH buffer. The values of the zeta potential adsorbents were quantified using (6) of* Smoluchowski* [23]. This was done by applying the integrated computational procedure through the Zeta Sizer Nano series equipment ZS from* Malvern 3600 Instruments*®. The electrophoretic mobility of the particles being measured was evaluated and converted into zeta potential values expressed in mV based on the pH:

(6) $$\begin{array}{c} Zp=\frac{4\cdot \pi \cdot v_{t}}{D_{t}}\times E_{m}, \end{array}$$

where *E*_*m*_ is electrophoretic mobility; *v*_*t*_ is suspending liquid viscosity (poise) at room temperature; *D*_*t*_ is dielectric constant; *Zp* is voltage (mV).

### 2.5. Adsorption Isotherms

In this study, two models of adsorption, Langmuir and Freundlich, were used to describe the BSA adsorption equilibrium of the adsorbents, as this interaction. In this case, the adsorption isotherms were obtained by soaking samples (0.0100 ± 0.0005 g) of adsorbents hydroxyapatite (HA), synthetic hydroxyapatite (HAS), and active babassu coal (ACB) in 1800 *μ*L BSA in different concentrations. Batches were obtained by testing, as described [10]. To samples containing adsorbents 800 *μ*L of the buffer solution (pH 4.0 and pH 7.0) was added followed by stirring for 1 h ± 25°C. Immediately various volumes of protein solution at different concentrations (0.5 to 7.0 mg mL^−1^) were added to complete the total volume of 1800 *μ*L, making each tube to have a different concentration, with a concentration gradient of BSA. The tubes containing the adsorbent and albumin were kept in a flurry of 20 rpm/3 h at 25°C. After this time, they were removed and centrifuged at 7500 g/15 min. An aliquot of the supernatant was taken for quantitation of protein in the fluid phase for direct reading at a wavelength of 280 nm (PG Instruments Ltd., T80 + UV-VIS Spectrometer). From the calibration curve previously determined, we found the concentration of protein adsorbed for mass of adsorbent by applying (1). The isotherms were adjusted in accordance with the models of Langmuir and Freundlich, as shown in the following, respectively:

(7) q=qm·C0kd+C,

(8) qs=KC1n,

where *q* is the concentration of protein adsorbed on the solid phase (g mg^−1^), *C*_0_ and *C* represent the initial and equilibrium concentrations (mg mL^−1^), *V* is the solution volume (mL), and *m* is the mass of the adsorbent material (g). *K*_*d*_ (mg mL^−1^) is the dissociation constant describing a measure of affinity or selectivity of the adsorbent for the protein balance of the adsorption reaction, *q*_*m*_ is the maximum adsorption capacity (mg g^−1^), *q*_*s*_ is the ability of adsorbent saturation, and *K* is the Freundlich constant [(mg g^−1^) (mg L^−1^) *n* ^−1^]. The exponent *n* is favorable if adsorption has value less than 1, indicating the tendency of the solute to migrate to the solid phase.

The Langmuir isotherm is a binding model which requires a dynamic balance between molecules and among those absorbed into the surrounding solution [24, 25]. The Freundlich model shows that the amount adsorbed in the process indefinitely increases with increase in concentration and has been characterized empirically as applicable to different systems [26].

## 3. Statistical Analysis

Performed in triplicate, with three repetitions, nonlinear models were fitted into the experimental data and kinetic adsorption isotherms, using the Gauss-Newton method. Adsorption data were fitted using two different models (Langmuir and Freundlich). The curves of the models were fitted into the experimental data using* SigmaPlot* 11.0. The models were evaluated according to the coefficient of determination (*R* ^2^) and the mean square error (RMSE) was calculated with

(9) $$\begin{array}{c} RMSE=\sqrt{\frac{\sum {\left(\overset{-}{Y}-Y\right)}^{2}}{N}}, \end{array}$$

where $\overset{-}{Y}$ is the variable predicted by the model, *Y* is the variable experimentally obtained, and *N* is the number of observations.

## 4. Results and Discussion

### 4.1. Kinetics of Adsorption

**Figure 1:**
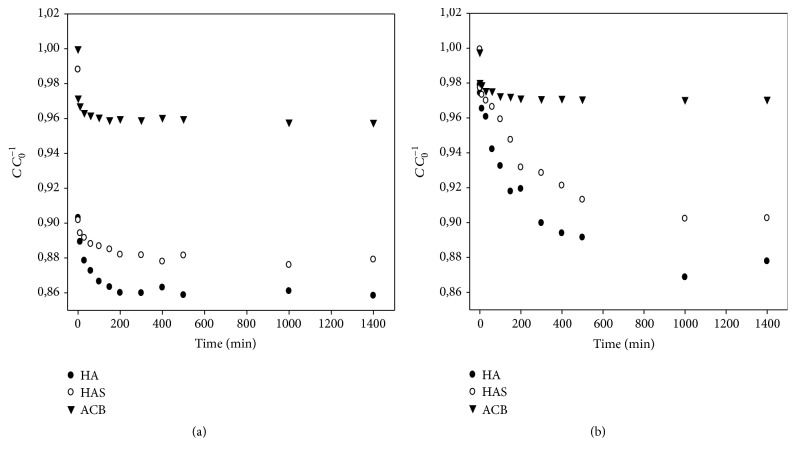
Kinetics of BSA adsorption on the surface of hydroxyapatite (HA), synthetic hydroxyapatite (HAS), and active babassu coal (ACB) at pH 4.0 (a) and pH 7.0 (b) at 25°C/24 h, with final concentration (*C*) and initial concentration (*C*_0_).

Figures 1(a) and 1(b) show the influence of time on the albumin adsorption of adsorbents (HA, HAS, and ACB) at pH 4.0 and pH 7.0. From the *C* *C*_0_^−1^ curves versus time (min), there is the retraction of the BSA concentration in the liquid phase with time.

It can be seen graphically that the time at which the adsorption capacity remains constant is close to 150 min for the HA and HAS in Figures 1(a) and 1(b); however for babassu saturation it is faster due to the presence of various functional groups at pH 4.0 and pH 7.0. Similar observation was reported in the literature for different adsorption process [12, 13].

**Figure 2:**
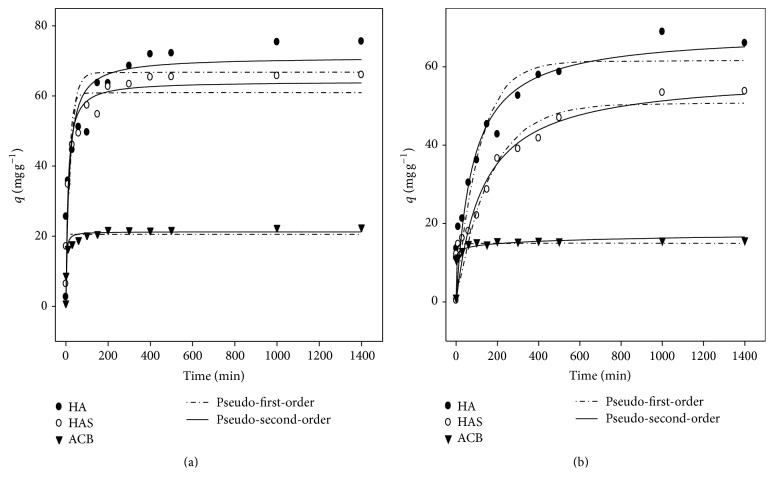
Kinetic pseudo-first-order and pseudo-second-order models for the adsorption of BSA on the surface of hydroxyapatite (HA), synthetic hydroxyapatite (HAS), and active babassu coal (ACB) at pH 4.0 (a) and pH 7.0 (b) and 25°C/24 h.

As illustrated in Figures 2(a) and 2(b), the kinetic pseudo-first-order and pseudo-second-order models are shown. Satisfactory adjustment of the models tested was obtained for the adsorbents in Table 1. From these results, it was found that the Pseudo second-order model fits best, based on average values of lower error residue of the square (RMSE) and the coefficients of determination (*R* ^2^) obtained.

The mass transfer rate in the adsorption process was slower for the ACB (*h* = 10.89 and 31.14 mg g^−1^ min^−1^). This could be due to the high molecular weight (69 kDa) protein of existing BSA and various functional groups in the active babassu coal, thus experiencing a rapid saturation of the pores. Regarding the HA and HAS, the process is faster possibly due to the existence of a high number of sites available for initial connections.

Table 1 shows the values of Lagergren [22] constants; *q*_*e*1_, *k*_1_, *k*_2_, and *q*_*e*2_ were calculated from (1), (2), and (3) of these models. Depending on the results found, the kinetic pseudo-second-order model presents chemisorption characteristics. The coefficients of determination (*R* ^2^) for the pseudo-first-order and pseudo-second-order models were high, and values were obtained as 0.8824 to 0.9813.

The balances in pH 4.0 and pH 7.0 of adsorbents (HA, HAS, and ACB) ranged from *q*_*e*,exp_ = 15.78 to 75.49 mg g^−1^. Yin et al. [18] evaluated the BSA adsorption kinetics of HA 18.5°C at pH 5.82 and 30°C at pH 7.0 with search results as *q*_*e*,exp_ = 59.06 and 41.49 and mg g^−1^; values were similar to those found in this work. Swain and Sarkar [15] studied BSA adsorption on hydroxyapatite at pH 7.4 and 37°C, having found values as *q*_exp_ = 28 mg g^−1^. A study with BSA on asset siriguela charcoal found values as *q*_*e*,exp_ = 29.35 to 58.26 mg g^−1^; *h* = 28.74/min to pH = 7.0; and temperatures between 20 and 40°C [12]. Studies with BSA have been conducted and the results obtained for the parameters *k*_1_ and *k*_2_ corroborate those found in this study. Kopac et al. [27] also reported *k*_1_ values close to those found in this work, being pH 4, *k*_1_ = 0.018, and 0.019/min at 20 and 40°C, respectively. The highest values of the rate constants, *k*_1_, pH 4.0, and pH 7.0, may be due to a change of the sites of the adsorbent [27]. From the results in this process, it is assumed that surface exchange reactions occurred until the sites were fully occupied, mainly by HA and HAS to the ACB which was difficult due to the presence of functional groups in the structure [28]. Pseudo-first-order and pseudo-second-order models showed a good fit to the experimental data. Generally the RMSE values of the Pseudo second-order model were lower, indicating that this model best explains the behavior of experimental data.

### 4.2. Quantification of Separation Efficiency (%) of Bovine Serum Albumin

The average results obtained for repetition of adsorption of bovine serum albumin in the test adsorbents tension and ACB at pH 4.0 and pH 7.0 and 25°C after 24 h are shown in Table 2.

In addition, the adsorption capacities (*q*) and the adsorption efficiency (Efic) for all adsorbents were obtained. It is observed that higher amounts of adsorption capacity and efficiency were obtained for the higher purity with hydroxyapatite (HA) and (HAS), synthetic at pH 4.0. This suggests that these adsorbents and pH are the most suitable for testing. It can be seen from the results that the various functional groups present on the activated carbon adsorption capacity are influenced. Similar observation was reported in the literature [13]. The values were higher than those obtained by Oliveira et al. [13]. Obtained values of 8.1, 26.2, and 25.4 mg g^−1^ and efficiency of 8.1, 26.1, and 25.5% at pH 3, pH 5, and pH 7, respectively, represented the adsorption of BSA on activated carbon from hog plum pits. Pereira et al. [12] studied the adsorption of BSA on activated carbon produced from bark and hog plum seeds and obtained values of 41.02 and 188.29 mg g^−1^ and efficiency between 21, 25, and 92.29%.

### 4.3. Zeta Potential (*Zp*)

**Figure 3:**
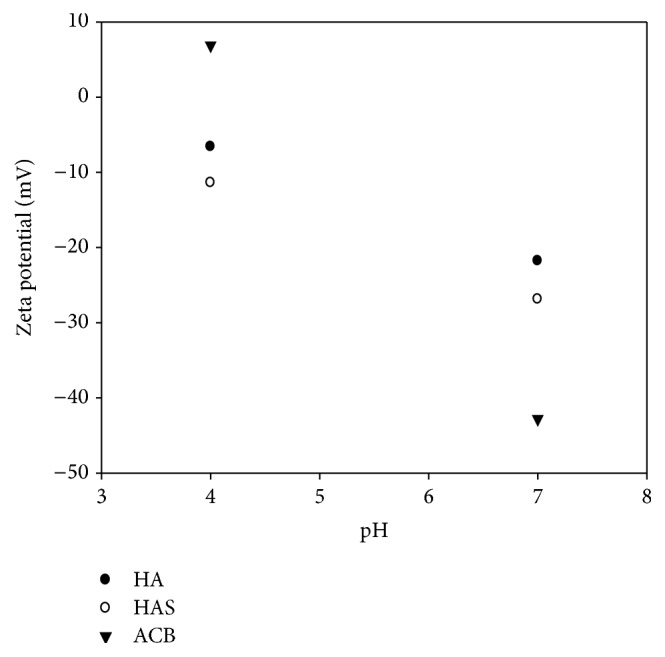
Zeta potential (*Zp*) obtained for the adsorbents hydroxyapatite (HA), synthetic hydroxyapatite (HAS), and active babassu coal (ACB) at pH 4.0 and pH 7.0 and 25°C.

The zeta potential measurements obtained for the adsorbents are shown in Figure 3. The results varied between −6.6 and −42.8 mV. It was observed that there was a greater increase (module) in pH 7.0. The values obtained for HA (−6.6 and −21.8 mV) and HAS (−11.4 and −26.9 mV) at pH 4.0 and pH 7.0 were justified in terms of the heterogeneous surface and multiple binding sites. In addition, the surface morphology and the degree of crystallinity were influenced in the production process by Norton et al. [29]. Similar results were obtained by Tercinier et al. [25], where *Zp* values were found to be hydroxyapatite (−11 and −28 mV) at pH 7.1. These results are due to the aqueous solution being rich in calcium ions (Ca^+^) and phosphate (PO_4_^−3^) of hydroxyapatite, which leads to the increase of electrostatic forces. This indicates the presence of adsorption acetate ions (Ac+) on the surface of HA and HAS. The diffusion of sodium ions (Na+) present in sodium acetate (NaAc) occurred as being dissolved, thereby binding to the surfaces by hypertension.

The literature presents various studies with hydroxyapatite. Osόrio [30] found *Zp* values of pH 3.0, pH 4.0, and pH 7.0; HA (0.0 mV; −10.0 mV and −30.0 mV) was obtained at pH 3.0, pH 4.0, and pH 7.0, respectively. In a study of carbon nanotubes and hydroxyapatite, values close to those were found in this work. Osόrio [30] analyzed the *Zp* of hydroxyapatite at pH 7.2 and found values in the range of −18.1 to −28.7 mV. The values supported this research. For the active babassu coal (ACB), Figure 3 showed values of +6.9 and −42.8 mV at pH 4.0 and pH 7.0, respectively. Studies show that the surface characteristics of coal (pore size and surface area) influenced the characteristics of loads [13]. The active carbon chemical structure, functional groups, such as heteroatoms, oxygen, and hydrogen plus inorganic components influence the adsorption [28]. These components influence the overall behavior of the surface charges, justifying the variation of the zeta potential. The same effect was observed by Valencia [31] who found +3 to −48 mV and Cottet [32] obtained +4 mV at pH 4 and −25 mV at pH 7 for active babassu coal.

### 4.4. Adsorption Isotherms

Adsorption isotherms were determined for the BSA protein in HA adsorbent, HAS, and ACB with pH 4.0 and pH 7.0 Experimental data were fitted with nonlinear model of Langmuir and Freundlich at 25°C. The plots show *q*, amount of protein adsorbed on the adsorbent (mg g^−1^) versus *C*, balance in protein concentration in the liquid phase (mg mL^−1^) in different adsorbents (HA, HAS, and ACB) with pH 4.0 to 7.0. The data of the isotherms showed that, from the tested concentrations, the obtained settings followed a similar result to the adsorption isotherm model in monolayers, indicating that the adsorption process occurred easily in active sites distributed uniformly on the surface of HA and HAS. For ACB adsorption, it was slower due to various functional groups [13].

**Figure 4:**
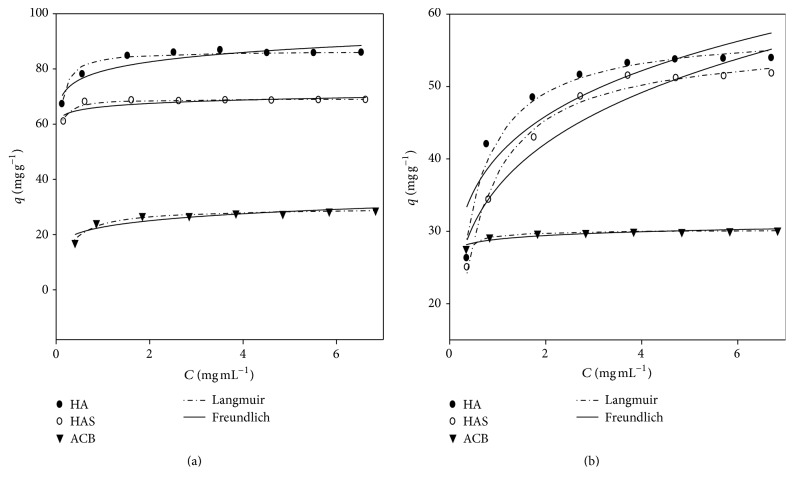
BSA adsorption isotherms on hydroxyapatite (HA), synthetic hydroxyapatite (HAS), and active babassu coal (ACB) at pH 4.0 (a) and 7.0 (b) at 25°C for adjustment of the Langmuir and Freundlich models. The symbols represent experimental data.

Figures 4(a) and 4(b) represent the BSA adsorption isotherms of the adsorbents hydroxyapatite (HA), synthetic hydroxyapatite (HAS), and active babassu coal (ACB) at pH 4.0 and pH 7.0.

All values of coefficient of determination (*R* ^2^) and RMSE were satisfactory; however the model of Langmuir *R* ^2^ presented higher and lower RMSE values for the three adsorbents in relation to the Freundlich model. It was observed that as the errors decreased (RMSE), the highest pH *R* ^2^ for each adsorbent was obtained. The values of Langmuir model indicate that this model best explains the behavior of experimental data. The Langmuir model is what best represents the adsorption of protein molecules in the adsorbents. This can be explained by the sensitivity of the Langmuir model in relation to the surface of the adsorbents. In the adsorption, the pH is an important parameter influencing the binding capacity for protein adsorption. The change in pH changes the charge distribution, net charge of BSA molecule, and the group in the contact regions beyond the molecular structure. Thus, the adsorption behavior reflects the nature of the physicochemical interactions of BSA and the active sites of the hydroxyapatite. The parameter settings of the isotherms were obtained from the correlation of experimental data. The Langmuir model showed better fits of the isotherms for the data in this study with *q*_*m*_ function, *R* ^2^, and RMSE (Table 3).

The pH values above 4.0 studied in this work showed the presence of negative charges (Figure 3). The binding capacity of hydroxyapatite with albumin showed a significant decrease with increasing pH. It is observed that at pH 4.0 the adsorbents have maximum adsorption capacity between *q*_*m*_ = 25.04 and 87.95 mg g^−1^ and pH between 7.0 *q*_*m*_ = 28.81 and 57.82 mg g^−1^ for the proposed models. This results in the BSA molecules becoming more compact, thus facilitating the adsorption process. There are electrostatic interactions between the cations (Ca^2+^) and anion (PO_4_^−3^) of HA with the anion (-COO^−^) and BSA NH4^+^ cation, thus changing loads [15].

At pH 7.0, the carboxyl groups dissociated more, resulting in increased negative charges on the adsorbent; so the BSA molecule with negative charge (pH > IP) led to lower retention in the active sites of hypertension. This explains the decrease in the adsorption of BSA for these adsorbents. The electrostatic repulsive force of HA, HAS, and BSA is generated and enhanced as the pH becomes more alkaline; it prevents the adsorption of BSA on the surface of HA and HAS.

The adsorption of BSA protein reaches equilibrium quickly at relatively low values of adsorption capacity (*q*_*m*_), about 25.04–36.18 mg g^−1^ for the ACB. This is due to the rapid saturation of active sites (pores) of coal. The functional groups present had an important role in low adsorption. They led to a lower retention in the active sites of hypertension, which explains the decrease in the adsorption of BSA for these adsorbents. This behavior was also observed by Oliveira et al. [13] for ACB, in a work performed with pH 3.0, pH 5.0, and pH 7.0. It demonstrates the behavior of functional groups present, which was found as *q* = 16.2 mg g^−1^, 19.1 mg g^−1^, and 26.2 mg g^−1^, respectively.

The BSA-HA and BSA-ACB complex Langmuir model shows dissociation constants (*K*_*d*_) estimated variation between 0.017 and 0.462 mg mL^−1^. These low values *K*_*d*_ < 1 indicate that the adsorption phenomenon was more favorable at pH 4.0, and balance was reached quickly. In the literature, values close to *K*_*d*_ 0.178 = 0.210 mg mL^−1^ were obtained for pH 4.0 [27]. In the Freundlich model, saturation capacity (*q*_*s*_) was higher at pH 4.0, with BSA migration tendency for the solute between *n* which showed 0.023 and 0.217. This indicates that the degree of heterogeneity of the adsorbent was better at pH 4.0. Similar results found by Kopac et al. [27] in the range *n* = 0.046 and 0.057 corroborate the job.

## 5. Conclusion

The kinetic analysis was best represented in the pseudo-second-order model with chemisorption characteristics. It was observed that the adsorption capacity of the adsorbent diminishes as the pH increases, indicating electrostatic linkages and functional groups contributing to this in order to reduce the adsorption of BSA. The Langmuir model showed better results, indicating greater homogeneity for HA and HAS. For the active babassu coal, the surface was more heterogeneous, especially oxygen linked to carbon atoms. The adsorbents HA and hypertension were more efficient in the separation of albumin process. Finally, it can be concluded that the separation of whey of bovine serum albumin is possible; however, there is still much to do in the contribution of this scenario, as shown in sequence some suggestions for future work:

- FTIR and NMR studies should be carried out after the synthesis of synthetic hydroxyapatite.
- Carry out specific purification procedures of protein obtained with worked adsorbents.
- Validate used purification procedures of protein obtained with worked adsorbents.

## Figures and Tables

**Table 1:** Kinetic parameters of BSA adsorption on hydroxyapatite (HA), synthetic hydroxyapatite (HAS), and active babassu coal (ACB) at pH 4.0 and pH 7.0 at 25°C.

Model	Parameter	pH					
		4.0			7.0		
		HA	HAS	ACB	HA	HAS	ACB
Pseudo-first-order	*q*_*e*,exp_	75.49	65.96	22.55	66.02	53.72	15.78
	*q*_*e*,calc_	66.75	60.97	20.59	61.56	50.70	14.83
	*k*_1_	0.0431	0.0659	0.5278	0.0090	0.0060	1.3026
	*R* ^2^	0.8824	0.9399	0.9571	0.9198	0.9404	0.9477
	RMSE	5.86	7.00	1.93	8.20	6.05	1.35

Pseudo-second-order	*q*_*e*,exp_	75.49	65.96	22.55	66.02	53.72	15.78
	*q*_*e*2,calc_	71.09	64.13	21.26	68.73	57.85	15.00
	*k*_2_	0.0010	0.0016	0.0241	0.0002	0.0001	0.1384
	*h*	5.05	6.58	10.89	0.94	0.33	31.14
	*R* ^2^	0.9310	0.9708	0.9813	0.9457	0.9546	0.9594
	RMSE	4.42	4.91	1.29	6.78	5.30	1.20

Quantity of BSA adsorbed on the calculated equilibrium: *q*_*e*,calc_ (mg g^−1^); BSA adsorbed amount at equilibrium experimentally obtained: *q*_*e*,exp_ (mg g^−1^); rate constants *k*_1_ and *k*_2_/min; time: *h*/min; average error of the residue of the square (RMSE).

**Table 2:** Concentration of the solution (*C*), adsorption capacity (*q*), and adsorption efficiency (efficiency) of bovine serum albumin (BSA) after 24 h at room temperature in pH (4.0 and 7.0).

Sample	pH	BSA		
		*C* (mg/mL)	*q* (mg/g)	Efic (%)
HA	4	0.774	67.5	74.2
HAS	4	0.942	61.8	68.6
ACB	4	1.878	32.9	37.4
HA	7	1.224	54.3	59.2
HAS	7	1.476	46.6	50.8
ACB	7	2.064	27.6	31.2

Hydroxyapatite (HA), synthetic hydroxyapatite (HAS), and active babassu coal (ACB).

**Table 3:** Parameters of adsorption isotherms of bovine serum albumin on the adsorbent hydroxyapatite (HA), synthetic hydroxyapatite (HAS), and active babassu coal (ACB) at 25°C in pH 4.0 and pH 7.0 for the Langmuir and Freundlich models.

Model	Parameter	pH × adsorbents					
		4.0			7.0		
		HA	HAS	ACB	HA	HAS	ACB
Langmuir	*q*_*m*_	87.95	68.26	36.18	57.82	55.90	30.90
	*k*_*d*_	0.030	0.017	0.034	0.031	0.462	0.382
	*R* ^2^	0.9832	0.9886	0.9811	0.9915	0.9884	0.9937
	RMSE	1.34	0.34	1.43	1.03	1.55	0.12

Freundlich	*q*_*s*_	81.55	65.84	25.04	42.16	36.34	28.81
	*n*	0.055	0.023	0.027	0.165	0.217	0.181
	*R* ^2^	0.9331	0.8449	0.9021	0.9043	0.9612	0.963
	RMSE	2.63	1.21	3.20	3.38	2.82	0.27

*q*_*m*_ is maximum adsorption capacity (mg g^−1^); dissociation constant is *K*_*d*_ (mg g^−1^) is depicting an affinity measurement. It is favorable if adsorption has value less than 1, indicating the tendency of the solute to migrate to the solid phase (*n*) dimensionless coefficient; the saturation capacity of the adsorbent is *q*_*s*_ (mg g^−1^); average error of the residue of the square (RMSE).
